# *‘It depends on who is asking and why they will use it’*: Access to male condoms in Timor-Leste

**DOI:** 10.1371/journal.pgph.0002409

**Published:** 2023-09-29

**Authors:** Helen Henderson, Helio Afranio Soares Xavier, Silvina Amaral Mendonca, Alexandrina Marques da Silva, Mariano da Silva, Rui Maria de Araújo, Meghan A. Bohren, Cathy Vaughan

**Affiliations:** 1 Marie Stopes Timor-Leste, Rua Belarmino Lobo, Bidau Lecidere, Dili, Timor-Leste; 2 Gender and Women’s Health Unit, Nossal Institute for Global Health, Melbourne School of Population and Global Health, The University of Melbourne, Carlton, Victoria, Australia; 3 MSI Asia Pacific, Melbourne, Victoria, Australia; 4 Independent Advisor, Dili, Timor-Leste; Western University of Health Sciences, CANADA

## Abstract

The uptake of male condoms remains markedly low in The Democratic Republic of Timor-Leste (Timor-Leste), an island nation in South-East Asia. To understand why, we conducted participatory and operational research about beliefs, understanding and access to male condoms from both a community and healthcare provider perspective. We held 14 participatory group discussions (PGDs) with 175 community participants (84 men, 91 women; aged 18–72) across seven municipalities (Ainaro, Baucau, Bobonaro, Dili, Lautem, Manufahi, and Oecusse) in 2019. We held individual in-depth interviews (IDIs) with 24 healthcare providers working in the same community catchment areas as the PGDs. Two counsellors, four doctors, fifteen midwives, and three nurses participated (16 women, 8 men; aged 25–56 years). Data were analysed using reflexive thematic analysis. PGD and IDI participant awareness, understanding and beliefs about male condoms were diverse. Male condoms were often discussed as something negative and taboo, and as something that is highly regulated and discouraged within society and the health system. However, many PGD and IDI participants also challenged this narrative by providing a more rights-based perspective about universal access to sexual and reproductive health information and services, including male condoms. Insights from our research have been used to inform programmatic decision-making in Timor-Leste, including health promotion and service delivery initiatives. Our findings can be further used to inform national health policy, healthcare provider training, and advocacy and communication work.

## Introduction

Modern contraceptive prevalence is low in The Democratic Republic of Timor-Leste (Timor-Leste), at only 24 percent [1]. One in four Timorese women have an unmet need for modern family planning [1]. Male condoms (hereafter referred to as ‘condoms’) are one of the least used methods in Timor-Leste, with a prevalence of less than one percent among all women (15–49 years) [1]. Low condom uptake is concerning, as condoms are a relatively cost-effective method of contraception, that when used consistently and correctly can prevent both pregnancy and sexually transmitted infections (STIs), including human immunodeficiency virus (HIV) [2,3]. Condoms can be used in conjunction with other modern contraceptive methods that are more effective at preventing unplanned pregnancy, but do not provide any protection against STIs (for example, hormonal methods of contraception or intrauterine devices). Condoms are also recommended as a backup method, when first starting contraceptive methods that may require time to be effective [3]. Indeed, worldwide, condoms are the second most commonly used method of all contraceptive methods (behind female sterilisation) [4]. Amongst methods of contraception specifically for men (condoms, vasectomy, and some natural family planning methods), condoms are the most commonly used method worldwide [4]. There are limitations in understanding data about condom use, however, including that men are not routinely asked about contraceptive use in national surveys, with data instead generated from responses from women, and often only married or in-union women [5]. This is the case for the most recent demographic data available for Timor-Leste (2016), with data about contraception use being collected only from women [1]. However, despite data limitations, it is clear that condom access and uptake is low in Timor-Leste.

Contraceptive method choice in Timor-Leste is improving yet remains skewed towards contraceptive injectables [1]. A skewed contraceptive method mix, in which 50 percent or more of contraceptive users rely on a single method (as is the case for contraceptive injectable use in Timor-Leste), can indicate insufficient availability of other methods or possible provider bias [6]. In reference to contraceptive services, provider bias refers to ‘*the practice of favouring some methods and discouraging others without medical rationale*, *due to their own prejudices about the method or its delivery system*, *and is often targeted to a particular client subset’* [7]. Given the skewed family planning method mix, provider bias may be an issue in Timor-Leste.

Challenges around supply chain management may also be a contributing factor to low condom uptake in Timor-Leste. Preliminary results presented from the 2018 national survey on family planning, reproductive and maternal health services showed only 37 percent of government health facilities had condom stock at the time of survey [8]. This is despite condoms being included as an essential item in the national basic primary healthcare package, that should be available at all government health facilities and mobile health services [9]. Condoms can also be purchased through the private sector. However, complications exist: in 2019, a private supermarket in the capital city Dili briefly displayed public signs indicating they no longer stocked condoms as they had been formally requested by an undisclosed authority to no longer sell them.

Timor-Leste has a low-level, non-generalised HIV epidemic, with HIV prevalence in the general population less than one percent in 2021 [10]. While more accurate estimates of HIV prevalence in Timor-Leste are urgently needed, available survey data shows that prevalence is increasing [11,12]. For example, HIV prevalence among pregnant women accessing antenatal care increased from 0.08% in 2016 to 0.3% in 2019 [13,14]. This is a 3.8 fold increase over just three years. Data about prevalence of other STIs are even more limited, due to the sporadic availability of STI testing and treatment across the country. Most health facilities provide only syndromic treatment for STIs. However, data showing increasing prevalence of HIV may indicate increases in other STIs also. Globally, condoms remain an important tool in the prevention of HIV and other STIs. Despite this, many public HIV prevention campaigns in Timor-Leste focus on abstaining from sexual relations rather than promoting the use of condoms [11].

Further, persisting patriarchal gender norms and sexual and gender-based violence against women continue to be significant public health issues in Timor-Leste, influencing a couple’s ability to use or negotiate condom use [15]. Only 41% of currently married women report being able to say no to their husbands when negotiating sexual relations, and only 25% report that they could ask their husbands to use a condom [1]. Challenges around the inability to negotiate consistent and correct condom use amongst men who have sex with men has also been described [16].

While previous research has explored women’s access to contraception and other sexual and reproductive health (SRH) services in Timor-Leste, limited evidence exists about access to and uptake of male methods of contraception, including condoms. We used an operational and participatory approach to explore both community and healthcare provider beliefs and understanding about male contraceptive methods in Timor-Leste. In this manuscript, we focus on our findings about the beliefs, understanding and access to condoms in Timor-Leste.

## Materials and methods

### Study context

Timor-Leste has an estimated population of 1.3 million, with a median age of 20.8 years [17]. Most people identify as Catholic (97.6%) and live in rural and remote locations (70.6%) [18]. Despite significant improvements in health outcomes since independence was gained in 2002, Timor-Leste still faces many social, political, and economic impacts left by centuries of Portuguese colonisation and decades of occupation by Indonesia, Japan and Australia [19,20]. Specifically for health, there are still significant challenges around sexual and reproductive health rights (SRHR), including, as described above, access to and uptake of contraceptive methods.

Terminology

We acknowledge the terminology ‘family planning’ and ‘contraception’ have different meanings and the need for rights-based language [21]. Contraception refers to the intention of an individual or couple to prevent pregnancy as a reproductive right [21]. Family Planning is the action of planning a family (if children are desired, how many, and when) [3,21]. However, reflecting the diversity of terminology used across global SRHR organisations, and the language most often used in Timor-Leste, both family planning and contraception are used interchangeably in our research and within this manuscript.

### Research approach

We used a participatory and operational approach to our research. Practically, this means we prioritised the involvement of people, groups and organisations most affected by the health problem (access to SRHR in Timor-Leste) in all stages of the research cycle (study design–data collection–data management–data analysis–communication and action of research findings) [22]. We also used participatory research methods in our study, including our use of body mapping and multilingual panel translation processes [23,24]. These methods provided flexibility, time, and scope for participants to engage in the research in ways they controlled and found acceptable.

We followed the Médecins Sans Frontières definition for operational research: *‘the search for knowledge on interventions*, *strategies and tools that can enhance the quality or performance of programs’*. Or more simply, ‘*the science of doing better’* [25]. We apply this to SRHR programming in Timor-Leste.

### Study design

Our study was implemented in collaboration between Marie Stopes Timor-Leste (MSTL), an SRH specialist organisation working in partnership with the Timor-Leste Ministry of Health, and The University of Melbourne. At the time of research design and data collection, the five multi-lingual and majority Timorese field research team members (HH, MS, HSX, SM, AS) were employed by MSTL in health program, management and service delivery roles. We developed research questions and methodology in collaboration with key stakeholders, including the National Health Institute of Timor-Leste, healthcare providers, and healthcare users in Timor-Leste. An author reflexivity statement reflecting on the dynamics of our research team and collaboration process is available in S1 Appendix.

We conducted participatory group discussions (PGDs) with community members and in-depth interviews (IDIs) with healthcare providers. Seven Municipalities (Ainaro, Baucau, Bobonaro, Dili, Lautem, Manufahi, and Oecusse) were selected for our study.

The PGDs, organised by gender, age and location involved: 1) body mapping activities about SRH, contraceptive methods, and contraceptive side-effects; 2) vignettes about a fictional Timorese couple; and 3) facilitated group discussion about family planning methods.

IDIs consisted of semi-structured open-ended questions followed by body mapping activities involving male and female body templates [24].

Tools and processes for PGDs and IDIs were piloted prior to the data collection phase. We report on our study using the Consolidated Criteria for Reporting Qualitative Research (COREQ) checklist (S2 Appendix) as a guide [26].

### Participants

PGD participants were purposively sampled based on age, gender, and location. Community members were eligible to participate if they were at least 18 years old and able to provide informed consent.

IDI participants were purposively sampled based on age, profession, and location. To participate, healthcare providers needed to be at least 18 years of age and currently working in a client-facing healthcare provider role, within the same community catchment area as the PGDs.

### Data collection–Participatory group discussions

We coordinated with community leaders (Xefe Suco/Village Chief) before the commencement of data collection. The community leaders shared information about the research with their communities and invited interested people to participate in the study. This prior engagement with community leaders was a sign of respect for local decision-making and helped ensure our research intentions were transparent and our presence welcomed and acceptable.

On arrival in a community, the field research team introduced themselves and explained the research activities to interested community members. We invited anyone interested to participate to then join us in the gender-specific groups and complete the informed consent process. The gender-specific small group work was conducted in private and quiet locations, usually at a local community centre or under a leafy tree. MS and HSX facilitated the male PGDs. HH and AS or HH and SM facilitated the women’s PGDs. Research activities were designed to support and encourage participants to engage with the research in their chosen language and communication style, and a total of 13 languages were used. The PGD research activities lasted for between two and four hours and were audio recorded. Audio recordings were verbally translated and transcribed to English using a multi-lingual panel translation process, described in detail elsewhere [23].

An education session about SRHR was offered to all participants in their gender-specific groups after the PGD research activities were complete. PGD participants were provided with refreshments (coffee, tea, snacks), five USD for time and transportation, and received SRHR information, service referrals, and condoms.

### Data collection–In-depth interviews

We coordinated with Municipality Health Directors from each study municipality before commencing data collection, to facilitate communication with facility-level health directors and healthcare providers. IDIs were conducted by HH, AS and MS. Most IDIs were conducted in a private room at the healthcare provider’s health facility. One IDI, on request by the participant, was held in a quiet area of a nearby restaurant. The IDIs lasted between 30 to 120 minutes in length, with a median duration of 65 minutes. IDIs were audio-recorded and transcribed verbatim in the language of data collection by the MSTL data entry officer. Transcripts were translated into English by a professional translator. At the end of the IDI, participants were provided with SRHR information, national health policies, health promotion resources, and if requested, condom stock.

### Data analysis

As a field research team, we collaboratively analysed data from the PGD and IDI activities using reflexive thematic analysis [27]. Practically, this involved reviewing, discussing, and reflecting on the data (body map images, transcripts, and reflexive field notes) as a group, over a series of analysis sessions. Spread out over a two-month period, these sessions involved increasingly deep and reflexive engagement with our data and each other. Our personal identities, experience and perspectives shaped how we engaged with the data as individuals and as a team, and how we generated initial codes. HH conducted two more rounds of data coding as an individual. We did a final review and discussion of generated codes as a complete field research team, through a series of online video calls (due to the COVID-19 restrictions in place at the time of analysis). Throughout the coding process, we grouped, split, and renamed codes as our understanding of the data evolved. This iterative coding process enabled us to deeply engage with the data and each other, and to gain rich analytic insights. We used these codes to collaboratively generate our research themes. Data were uploaded into NVivo [28] for storage and data management.

### Ethical considerations

Ethics approvals were provided by the National Health Institute of Timor-Leste (1168MS-INS/DE/DEP/V112019), The University of Melbourne (1954731.1), and MSI Reproductive Choices in the United Kingdom (020–19). A plain language statement was provided to all participants in Tetun. Written and verbal consent was provided by all participants. We emphasised their responses were confidential and de-identified. Pseudonyms were given to all participants during the PGD activities. We practiced active informed consent throughout the research process, reminding participants they could participate in a way they felt comfortable with and were able to exit the research at any time. A distress protocol was in place to prevent, mitigate and respond to potential participant distress during the research. All healthcare providers approached to take part in the study consented to participate in the research. All community members who decided to join the gender specific PGD groups consented to participate in the research. Two female participants from different PGDs left before the end of activity three, to return home for child-care duties. We provided both participants with SRHR information, referrals and remuneration before leaving. Members of the National Health Institute of Timor-Leste ethics board conducted routine quality monitoring of the field research on two separate trips, observing three PGDs and two IDIs in two different municipalities (Bobonaro and Manufahi).

## Results

We held 14 PGDs with 175 community participants (84 men, 91 women; aged 18–72) across seven municipalities between August-December 2019 (Table 1). Participants spoke thirteen languages during the PGD sessions (Tetun, Portuguese, Indonesian, English, Tetun Terik, Baikenu, Bunak, Fataluku, Kairui, Kemak, Lakalei, Makasai, and Mambai).

**Table 1:** Socio-demographic summary of community members taking part in the participatory group discussions.

	Younger women’s group (n = 43)	Younger men’s group (n = 43)	Older women’s group (n = 48)	Older men’s group (n = 41)	Total participants (n = 175)
**Municipality**					
Ainaro	5	9	7	8	29
Baucau	5	4	5	5	19
Bobonaro	7	6	10	8	31
Dili	6	4	6	3	19
Lautem	9	6	7	5	27
Manufahi	3	6	7	6	22
Oecusse	8	8	6	6	28
**Location–n (%)**					
Rural	13 (30%)	19 (44%)	29 (60%)	23 (56%)	84 (48%)
Urban	30 (70%)	24 (56%)	19 (40%)	18 (44%)	91 (52%)
**Age–median (range)**	19(18–26)	23(18–33)	33(19–72)	31(19–59)	25(18–72)
**Education–n (%)**					
No formal education	1 (2%)	1(2%)	0 (0%)	3 (7%)	5 (3%)
Primary school	0 (0%)	0 (0%)	9 (19%)	6 (15%)	15 (9%)
Secondary school	35 (81%)	31 (72%)	37 (77%)	23 (56%)	126 (72%)
Vocational	3 (7%)	0 (0%)	0 (0%)	1 (2%)	4 (2%)
University	4 (9%)	11 (26%)	2 (4%)	8 (20%)	25 (14%)
**Marital status–n (%)**					
Married	2 (5%)	6 (14)	36 (75%)	34 (83%)	78 (45%)
Single	39 (90%)	37 (86)	2 (4%)	7 (17%)	85 (48%)
Divorced	1 (2.5%)	0 (0)	2 (4%)	0 (0%)	3 (2%)
Living with partner	1 (2.5%)	0 (0)	8 (17%)	0 (0%)	9 (5%)
**Employment–n (%)**					
Student	23 (53%)	12 (28%)	0 (0%)	2 (5%)	37 (21%)
Unpaid household	4 (9%)	0 (0%)	29 (65%)	0 (0%)	33 (19%)
Volunteer	14 (33%)	18 (42%)	11 (23%)	12 (29%)	55 (31%)
Agriculture	0 (0%)	5 (12%)	0 (0%)	19 (46%)	24 (14%)
Private sector	0 (0%)	0 (0%)	1 (2%)	4 (10%)	5 (3%)
Government sector	2 (5%)	3 (7%)	7 (10%)	4 (10%)	16 (9%)
Unemployed	0 (0%)	5 (12%)	0 (0%)	0 (0%)	5 (3%)
**Number of living children—median (range)**	0 (0–1)	0 (0–4)	3 (0–8)	2 (0–9)	1 (0–9)
**Self-identified kinship system–n (%)**					
Matrilineal	10 (23%)	7 (16%)	17 (35%)	10 (24%)	27 (25%)
Patrilineal	33 (77%)	36 (84%)	30 (63%)	31 (76%)	63 (74%)
Other	0 (0%)	0 (0%)	1 (2%)	0 (0%)	1 (<1%)

We held IDIs with 24 healthcare providers working in the same catchment areas as the PGDs. Two counsellors, four doctors, fifteen midwives and three nurses participated (16 women, 8 men; aged 25–56) (Table 2). IDI participants had diverse educational backgrounds, having completed their pre-service clinical training within Timor-Leste and internationally (Malaysia, Indonesia, Cuba, Australia, and Pacific Island nations). IDIs were conducted mostly in Tetun. Two IDI participants spoke in a mix of Tetun and Indonesian, and one in a mix of Tetun and English. Reflecting the linguistic diversity of Timor-Leste and the diverse educational backgrounds of the healthcare providers, most IDI participants also incorporated words or phrases, especially clinical terms, in English, Indonesian, Portuguese, and Spanish.

**Table 2:** Sociodemographic summary of healthcare providers participating in in-depth interviews.

	Total (n = 24)
**Gender** Women	16
Men	8
**Median age (age range)**	36.5 (25–56)
**Location** Rural	9
Urban	11
Mobile (visiting rural and urban sites)	4
**Municipality** Ainaro	3
Baucau	3
Bobonaro	3
Dili	7
Lautem	2
Manufahi	3
Oecusse	3
**Profession** Midwife	15
Nurse	3
Doctor	4
Counsellor	2
**Professional experience as a healthcare provider (years)**	
<1	1
1–5	5
6–10	8
11–15	3
16 +	7
**Health facility level** Hospital	3
Community health centre	6
Health post	4
Mobile outreach at public and private health facilities	9
Counselling site	2
**Marital status** Married	20
Single	1
Divorced	1
Living with partner	2
**Number of living children–median (range)**	2 (0–6)
**Self-identified kinship system**	
Matrilineal	7
Patrilineal	16
Other	1

We present our research findings under the following themes: 1) information, misinformation, and knowledge about condoms; 2) sexual health versus reproductive health; 3) complex gender and social norms around condom use; 4) roles and responsibilities in the provision of condom services; and 5) inconsistencies in condom supply and distribution.

### 1. Information, misinformation, and knowledge about condoms

PGD and IDI participants described low awareness and limited knowledge about condoms across Timor-Leste.

*“Condoms exist in Timor but nobody knows about them*. *Sometimes even at the health facility the information won’t be clear*, *or they just won’t talk about it”* (Men’s PGD participant, 25 years, rural location)

Many PGD participants were aware of condoms and were able to identify and describe them with various levels of detail. Some PGD participants were only aware of the name, while others could accurately describe the function and use of the condom. Among PGD participants aware of condoms, there was however widespread misinformation about condom function, especially amongst younger participants, and those without children.

*“Men use condoms so the penis won’t stand up*. *The condom stops the penis standing up so it can’t enter the woman”* (Women’s PGD participant, 21 years, urban location)

Many PGD participants also described incorrect use of condoms. For example, delayed condom use, in which the condom is put on after sexual relations have started but just prior to expected ejaculation. Many PGD participants also described inaccurate perceptions about the efficacy and side-effects of condom use. Table 3 shows examples of side-effects of condom use described by PGD participants, including that condoms spread disease and kill babies.

**Table 3:** Side-effects of male condom use, as identified by PGD participants.

Accurately identified or described potential side effects of male condom use	Inaccurately identified or described side effects of male condom use
○ Risk of an allergic reaction ○ Risk of pregnancy (not 100% effective)	○ Causes disease ○ Spreads disease ○ Kills babies ○ Causes promiscuity ○ Causes cheating or infidelity ○ Erectile dysfunction/impotence ○ Is 100% effective at preventing pregnancy

Many PGD participants also spoke about a reduction in sexual pleasure, almost exclusively in regard to male sexual pleasure, as a concerning side-effect of condom use. Two healthcare providers participating in the IDIs also spoke about condoms reducing sexual pleasure.

*“It* [condom] *decreases pleasure*. *I always say condoms are not good because when having sex*, *two bodies should touch each other”* (Male IDI participant, health counsellor, 43 years, urban location)

### Sources of information about SRH and condom use

PGD participants identified numerous sources of information about SRH and condoms, based on their gender and age groups (Table 4). Participants in the men’s PGD identified more sources of information than participants in the women’s PGD. The younger groups identified more sources of information than the older groups.

**Table 4:** PGD identified sources of information about condoms and sexual and reproductive health.

Younger women’s group	Younger men’s group	Older women’s group	Older men’s group
Boyfriend Female friends* Midwife* Doctor* Health centre Traditional healer Mother* Auntie Older sister MSTL youth hotline Health organisations Teacher Internet Social media Television	Girlfriend Male friends Female friends Mother Father Older brother Midwife Nurse Doctor Health centre MSTL youth hotline Health organisations Teacher Internet Social media Television Radio Newspapers	Husband Female friends Midwife Doctor Health centre Traditional healer	Wife Male friends Female friends Midwife Nurse Doctor Health centre Pharmacist Traditional healer Health organisation

*Identified by participants as both helpful and possibly harmful sources of information about preventing pregnancy or planning a family.

Across the PGDs, healthcare providers were described as an important source of information about condoms and SRH more generally. However, PGD participants of all genders and ages also identified fear and shyness in approaching a healthcare provider or visiting a health centre about SRH or condom needs, especially for young and unmarried people.

*“Not many people will go ask about this* [condoms], *people will feel shy to go to the health facility and ask for this”* (Men’s PGD participant, 23 years, rural location)

Some participants identified that sources of information about condoms and SRH also posed risks for the individual asking. For example, while mothers, midwives and doctors were identified as being supportive and reliable sources of information for younger women, they were also identified as possibly harmful information sources. Possible ramifications for a young woman asking midwives and doctors for information about SRH or condom use included shame, discrimination, and lack of confidentiality. Possible ramifications for a young woman asking her mother for information about contraception and SRH included shame, isolation, withdrawal of financial support and physical violence within the family.

*“It’s complicated*. *Some mothers are supportive*, *some aren’t*. *Maybe they can kill you*. *It’s especially harder for the girl child*. *Some brothers will beat up the girl for talking about this* [contraception]*”* (Women’s PGD participant, 18 years, rural location)

Fathers and older male family members were not identified as possible sources of information about SRH or condoms by any younger female participants. Fathers were identified as possible sources of information for young men. However, participants discussed that in reality this was difficult and would rarely happen due to embarrassment, shyness, or fear.

*“Young men*, *they are not getting information from their fathers and they are too afraid and shy to ask*. *They won’t ask their father or their teachers and neither will young women”* (Men’s PGD participant, 23 years, urban location)

Many of the older PGD participants also spoke about communication challenges relating to SRH and contraception within the child-parent relationship.

*“As a parent*, *if my child holds this* [condom], *I will be angry with them”* (Women’s PGD participant, 36 years, rural location)

IDI participants identified similar sources of community-facing information about condoms and SRH as the PGD participants. This included self-identifying healthcare providers as important and reliable sources of information. However, most healthcare providers participating in the IDIs also shared that young or unmarried people had never asked them about contraception or visited their health facility. While most said they would share information about contraception and SRH with a young or unmarried person if they were ever to be asked, two IDI participants said they would not share that information with a young or unmarried person, even if they were asked. Instead, these participants explained they would only share information about contraception with married couples, due to both personal and professional beliefs and understanding (discussed in more detail below). In contrast, four healthcare providers participating in the IDIs described regularly providing information about contraception and SRH to young and unmarried people as part of their typical duties. Further, they described their role and identity as healthcare providers beyond simply fulfilling their duties at a health facility (attending clients that come to them) and described their role in actively sharing information about condoms and SRH in the community, and in their circle of friends, family, and neighbours.

While both PGD and IDI participants described difficulty speaking about condoms openly, some IDI participants reflected on the progress made in making information more readily available.

*“I think the problem with condoms is not as taboo as it was during Indonesian times* [1975–1999]. *Back then it was very difficult to talk about it*, *so in my opinion we have made progress*. *A lot of people were embarrassed and had limited knowledge… It’s not as taboo as it used to be*, *but yes*, *young people are still afraid*, *they are afraid to be seen and maybe are scared and embarrassed of the health providers”* (Female IDI participant, midwife, 46 years, urban location)

### 2. Sexual health versus reproductive health

Most PGD participants with prior awareness about condoms discussed condom function as being to either prevent pregnancy or to prevent the spread of STIs or disease. Some PGD participants disputed that condoms were a type of family planning method at all, instead believing that only women could use methods of contraception to prevent pregnancy.

*“We can use condom but not family planning*. *Where would we put it in our body because men can’t get pregnant and give birth”* (Men’s PGD participant, 42 years, rural location)

There was limited discussion by most IDI participants about condoms being used in conjunction with other methods of contraception, or as a backup method as part of standard contraceptive care practice.

### Condoms and understanding about HIV and STIs

Only some PGD participants were able to describe the dual function of preventing pregnancy and the spread of STIs. While most of the healthcare providers participating in the IDIs identified the dual function of the condom, it was still mostly discussed in the context of preventing STIs.

*“Sometimes because of STIs people need to use condoms… because the benefits of condoms… condoms can be useful*. *It doesn’t mean everyone should use them though*, *no*. *Condoms should only be used for protection from STIs”* (Female IDI participant, midwife, 39 years, rural location)

For both PGD and IDI participants, most of the discussion about STIs focused on HIV and only a few participants discussed other STIs. There was substantial misinformation and confusion about what STIs are and how they are transmitted. For example, many PGD and some IDI participants described STIs as something that can always be physically seen on an individual. Some disorders and diseases that are not sexually transmitted were also described by some PGD participants as being types of STIs, for example, epilepsy and diabetes. Further, STIs were most often described by PGD participants as being transmitted from women to men:

*“In this community many women have disease and they will give it to men who are not careful”* (Men’s PGD participant, 30 years, rural location)

Most IDI participants considered that the rates of STIs in Timor-Leste were a significant challenge and condoms could help address this.

### Experiences in providing family planning and STI services

All IDI participants described providing varying levels of family planning care as part of their professional responsibilities. However, only a few healthcare providers described being trained or having competency for other sexual health services, including STI counselling, testing and treatment.

*“If the patient comes with an STI… we always check to see if they have an STI when they come to have FP* [family planning]. *This is because there are some FP methods that we can provide straight away*, *even if the person has an STI*, *we can provide it*. *But some FP methods can’t be given when there is an active STI infection*. *We need to treat the STI first*, *so we offer them another FP method or ask them to come back”* (Male IDI participant, nurse, 26 years, urban location)

Several midwives participating in the IDIs discussed conducting regular HIV and syphilis tests, as part of standard antenatal care.

However, most IDI participants described not being able to provide any STI services, including counselling and testing for HIV and syphilis. This was typically because STI services were considered separate from the responsibilities of contraceptive care, or due to a lack of provider training or facility resources.

*“We are not providing HIV tests here yet*. *I know we should provide them for the pregnant women but we haven’t received training yet so we can’t offer this”* (Female IDI participant, midwife, 31 years, rural location)

In larger health facilities, some IDI participants described being able to refer clients with STI needs to other healthcare providers within the same facility. In these instances, the family planning provider (typically located within maternal health services) could refer clients to a specific STI provider, who was described as being based in a different part of the health facility and often available on different days to family planning services. Further, most healthcare providers participating in the IDIs described family planning as a service specifically for mothers and pregnant women.

*“Usually STI and FP programs are separate*. *They might refer patients to each other but it’s a separate service*. *Different health providers attend STI patients*, *they have different training and a different focus to us*. *We provide family planning*, *we support mothers”* (Female IDI participant, midwife, 36 years, urban location)

While some of the challenges around combining sexual health and reproductive health services were structural (provider training, facility resources, task allocation), challenges around the gendered nature of SRH service provision were also raised by participants. For example, several IDI participants raised concerns and examples of the sexual harassment of female healthcare providers when attending to male clients with sexual health needs.

*“For male STI clients with female providers*, *in the past we have had cases of men harassing the female providers*. *They will ask the female provider to touch their penis*. *So we try to have a male provider attending a male STI client or if it’s a female provider have another person in the room so they are not alone”* (Male IDI participant, nurse, 26 years, urban location)

Some PGD participants also raised concerns about receiving sexual health services from a healthcare provider of a different gender. Some women’s PGD participants described cancelling or delaying an SRH service if a female provider was not available, due to feeling shy, embarrassed, or scared to be attended by a male provider. Several women’s and men’s PGD participants raised concerns about a woman’s risk of violence from her male partner if he became aware she had been attended to by a male healthcare provider. Several male PGD participants also raised concerns about being judged or seen as abusive when receiving sexual health care from a female healthcare provider.

*“If a man meets with a woman* [provider] *she will feel like we are harassing her*. *This is a problem*. *Some women providers will explain truthfully to us about sexual and reproductive health*, *but she will also think it’s sexual harassment and judge us”* (Men’s PGD participant, 39 years, rural location)

### 3. Complex gender and social norms around condom use

There was substantial diversity in how participants described condoms and the type of people who use condoms. While some PGD and IDI participants discussed the benefits of condoms and believed that anyone can use them, most PGD and IDI participants believed that only certain types of people should be able to access and use condoms, under certain circumstances. Almost all PGD and IDI participants identified that it was more socially acceptable for men (young, single or married) to access and use condoms than women (young, single or married). Women, especially young women, faced stigma, discrimination, or violence for just talking about or carrying a condom. Further, many IDI and PGD participants described women, especially young women, who use condoms as being sexually active and therefore unfaithful, promiscuous, or immoral.

*“Carrying a condom would create a problem for a single woman*. *People will think she uses a condom to have sexual relations”* (Women’s PGD participant, 26 years, urban location).

*“If a woman asks for a condom it means that she is thinking about free sex* [sex before marriage] *… that she knows how to use it and yeah… it’s not good for women*, *especially young women”* (Female IDI participant, midwife, 39 years, urban location)

Some PGD participants believed only married people could access condoms, while others believed it was only for young and unmarried people, especially young men. Although most of the group discussions about who uses condoms were focused on restrictive and negative descriptions, some participants spoke about the benefits of condoms. In these instances, people who used condoms were described as responsible, respectful, and loving people. Conversely, some PGD participants spoke about the benefits of using condoms while still describing the people who use condoms as *nakar-teen* (naughty), *malkriadu* (disrespectful), or *aat* (bad).

Using condoms during same-sex relations between men was discussed by several PGD and IDI participants, in the context of HIV and STI prevention. Same-sex relations were mostly discussed as covert and not accepted within society.

*“I’m sure we have gay patients but it’s not something we suspect*, *it’s not what we see during the consultation because it’s common in Timor for people to feel shy or scared*. *They won’t talk about it and when we look at them*, *we don’t know they are gay*. *It’s not very open here yet*, *people live in secret*. *No one has come and said to me they like men and need condoms”* (Male IDI participant, doctor, 35 years, urban location) **Trust and respect when using condoms**

Many PGD participants described condom use as something that only happens in relationships that did not have love, trust or respect. Condom use was most often associated with sexual relationships with female sex workers, with a woman outside of marriage (infidelity), or any relationship that was considered casual or non-serious. In all these cases, the use of condoms was required as women were described as being *aat* (bad) or *foer* (dirty).

*“They* [condoms] *are made to protect people’s health from sickness*. *And most sickness comes from women*. *Sometimes*, *because sickness comes from the woman*, *a man who doesn’t have disease will go and have sexual relations with a woman and disease will then go to him*. *He will get disease from a woman*. *In the future if you have a family*, *you can transmit disease from the dirty woman to your wife”* (Men’s PGD participant, 28 years, rural location)

Several healthcare providers participating in the IDIs echoed this sentiment about using condoms only when there is a lack of trust between sexual partners, and the impacts this can have on condom negotiation and decision-making within a relationship.

*“People should use it* [condoms] *when they need it*. *If they have sexual relations with someone they don’t trust*, *then they need to use condoms or risk getting an STI”* (Male IDI participant, nurse, 26 years, urban location)

*“Often it’s the wives that don’t want to use this* [condom]. *First is that they think a condom is for cheating so they will question why they need to use it*. *They might say ‘I am not a bad woman’*.*”* (Male IDI participant, nurse, 36 years, urban location)*Selingkuh* (infidelity or cheating) or men having multiple sexual partners was described as common practice by many PGD and IDI participants. Many PGD participants described that when a male partner cheated on his female partner, condom use with the other person was a sign of respect to protect the wife or female partner from disease. Importantly, although some PGD and IDI participants described condoms as encouraging or facilitating cheating behaviours and sexual relations more generally, most acknowledged that sex, including sex outside of marriage, will happen with or without access to condoms.

*“Many people think if condoms are more available*, *in all places*, *then people will have free sex* [sexual relations with more than one partner]. *They are scared that young minors will have sex and that men will cheat on their wives… sex happens already*. *Sex will continue to happen with or without condoms*, *so better we promote condoms to prevent early pregnancy and disease”* (Female IDI participant, midwife, 45 years, urban location)

### The influence of religion and culture

The important role of religion and religious leaders influencing social norms around SRH and access to condoms was discussed frequently by PGD and IDI participants.

“*No, single men cannot use condoms. It will destroy somebody’s daughter and give sickness to people’s daughters*. *Cannot*. *God says no. To use a condom, need to wait until married*” (Men’s PGD participant, 32 years, urban location)

*“Many health providers are also included in the church*. *They will never give condoms because they are part of the church and believe God*, *believe the church doesn’t support condoms*, *so they won’t provide them”* (Male IDI participant, nurse, 56 years, urban location)

More commonly, participants discussed religious and church leaders communicating anti-condom sentiments within the community.

*“Some people think when using a condom*, *it’s a thing that can destroy a baby*. *Some people talk like this*. *The church says it can kill people”* (Female IDI participant, midwife, 24 years, rural location)

*“Babies are thrown away here*, *you hear it on the news and in social media and everybody talks about it and often the priests blame that on NGOs that provide condoms and information about condoms*. *They put blame on the young girl and the health workers*. *They say condoms encourage free sex* [sexual relations outside marriage] *and because of that*, *babies are thrown away”* (Female IDI participant, midwife, 36 years, mobile location)

The importance of proactively engaging with the church at the local level was also highlighted by many IDI participants as vital for the effective delivery of SRH and contraceptive services. Some healthcare providers participating in the IDIs described difficulties they faced when not having local support from the church. This included healthcare providers having to face personal or professional critique about their values or position in society.

*“Anytime we try to share information or hand out condoms we need to go and ask permission from the priests*. *So we ask them before we do anything*, *like provide services or speak with the community*. *If not*, *the priest gets up every Sunday during mass and makes announcements ‘this institution*, *from this NGO*, *they came to hand out condoms so young people can have sexual relations’*.*”* (Male IDI participant, nurse, 56 years, urban location)

While the influencing role of religion and the church was typically discussed by IDI and PGD participants as being a challenge or barrier to accessing SRH and family planning services, some participants also spoke about the enabling role that religion and the church plays. However, this was discussed as being highly localised and dependent on individuals.

*“…Some priests are supportive*, *and understand a condom can prevent early pregnancy and disease*, *they understand it can save lives*, *the young women’s life and the baby’s life*. *Some priests are not supportive and say that condoms are against religion*, *against culture in Timor*. *So it’s really hard*. *It depends on the priest and the location*, *you never know*. *I think it is important for the church and government to sit together and talk about this*, *have a consistent plan to help the health of people in Timor*, *because honestly at the moment*, *it’s hard”* (Female IDI participant, midwife, 36 years, urban location)

### 4. Roles and responsibilities in the provision of condom services

Healthcare providers were identified as important gatekeepers to contraceptives by most PGD and IDI participants. Some IDI participants described providing universal access to family planning services, including condoms. However, most described limiting access based on a clients age, sex, gender, marital status or parity. The type of restriction and reasoning for the restriction varied significantly amongst the IDI participants. For example, several participants shared that they would provide condoms to unmarried men but not unmarried women because they considered while unmarried men could be sexually active, unmarried women should not be. Others described restricting access to unmarried people, married people, single people, young people, women, or people who attend a consultation alone.

### Tension between personal provider beliefs and quality of care delivered

Inconsistencies in service provision were identified by many IDI participants as being based on the personal beliefs or values of a healthcare provider.

*“I don’t know if many health providers*, *especially midwives*, *I don’t know if they are ready to give out condoms or not*… *some of the midwives here*, *they have morals that are different to health… it’s like… I don’t understand*. *They are responsible for family planning services here but they don’t provide”* (Male IDI participant, doctor, 35 years, urban location)

PGD participants reinforced these service provision inconsistencies. Most PGD participants described the ability to receive SRH services as being dependent on the personal belief and decision of the healthcare provider.

*“Health providers will give it* [condom] *when the objective is good*. *It depends on who is asking and why they will use it*. *The provider will ask lots of questions before giving it or not”* (Men’s PGD participant, 23 years, rural location)

Several healthcare providers participating in the IDIs also spoke about their oath and responsibility as healthcare providers to provide universal access to health services, regardless of their personal beliefs.

*“For some young people… I don’t want to give [*condoms*] to them because they will have sexual relations randomly*, *promiscuously*, *but these young people*, *these are also the people I need to give condoms to*, *to prevent them from giving diseases to women*, *or early pregnancy*. *I would prefer not to give*, *but it’s my responsibility as a health provider to give these*. *I have to give*, *I’m a midwife”* (Female IDI participant, midwife, 31 years, rural location)

### Disconnect between policy and practice

The knowledge and understanding of relevant SRHR policies and guidelines was diverse among IDI participants. Several providers participating in the IDIs reported not knowing if national health policies and guidelines existed about family planning or SRHR, or where such documents could be found. Some believed that national family planning policy documents did exist, but that they were confidential and high-level documents, only accessible to health directors and health facility managers. About a third of IDI participants were aware of national health policies related to family planning and SRHR. However, their understanding about what the policies allowed varied significantly, and was often believed to be highly restrictive. Perceived restrictions in access to family planning services were based on gender, age, civil status, and number of children. These restrictions were often described in regard to the law.

*“The thing is… the thing is that providers are afraid to attend young people because of the law*. *They don’t attend young people because the law doesn’t allow it*, *the church doesn’t allow it*. *Even if she needs it and she asks for it*, *they can’t give it to her*. *The government should think about creating a law so that women of reproductive age can get this information and use FP so they can prevent*, *they can prevent pregnancy and then babies won’t be thrown away”* (Male IDI participant, nurse, 56 years, rural location)

Conversely, some IDI participants did believe that family planning services could be provided to anybody who needed them. Participants with awareness and correct knowledge of national family planning policies described with frustration the inability to follow the policy due to restrictions or misunderstandings enforced within the health facilities or local areas in which they work.

*“I think the government policy is good*, *it promotes FP*. *The issue is that many people don’t know the real policy and get confused about what is allowed*. *They don’t read the policy*, *they just hear rumours or myths and provide based on their personal feelings and values”* (Male IDI participant, nurse, 26 years, urban location)

### 5. Inconsistencies in condom supply and distribution

Most PGD and IDI participants described overall low availability of condom stock across Timor-Leste, from both public and private sector sources. In particular, participants described the challenges of procuring condoms in rural and remote locations. Many PGD participants also described the need to procure condoms quietly or in secret, through the private market. This need for privacy and confidentiality was at odds with how many PGD and IDI participants described access at public health facilities. Most participants described the need for individuals to record their name, marital status and contact details before getting condoms at a public health facility. The need to collect this information was described by many IDI participants as a way to control or monitor condom use and ensure that only certain types of people could access the service (for example, married people). IDI participants described healthcare providers and facility managers stopping initiatives to provide easy and anonymous access to condom stock at a facility level, for example by removing condom stock from health facility bathrooms.

*“We don’t put them* [condoms] *in the toilet anymore*. *We put some in there before and people came and took it so quickly and we don’t know who*. *So it’s better we hold them*. *If they come and ask*, *we will give it to them but until now*, *they never come and ask”* (Female IDI participant, midwife, 46 years, urban location)

Most healthcare providers participating in the IDIs described a lack of consistent condom stock as a significant challenge to meeting client demand and increasing condom uptake across Timor-Leste. Some IDI participants reported not having condom stock at their health facilities for several months to three years.

*“We haven’t had condom stock for approximately a year now… I don’t know why… I don’t know*, *maybe we request condom stock and people don’t want to give it or maybe they don’t have any stock in Dili*? *I don’t know*, *but patients here ask for it and we can’t give it… A lot of the time*, *men came here a lot and ask us ‘Are there condoms or not*?*’*. *There are a lot of people asking this but no stock”* (Male IDI participant, doctor, 35 years, urban location)

However, not all providers considered the lack of condom stock a concern or a priority in their work.

*“If we have condoms we provide*. *If we don’t have any*, *we don’t give*. *It’s not a priority”* (Female IDI participant, midwife, 29 years, urban location)

## Discussion

Due to the unique and important ability of condoms to prevent both STIs and pregnancy, significant research has been conducted about the access and uptake of condoms globally [29,30]. Our findings provide new and contextual understandings about condom access and uptake in Timor-Leste, which also support and add to the global body of evidence. For example, our research indicates a disconnect between current programming for sexual health and for reproductive health in Timor-Leste, realised in how SRH services are understood and delivered. Our study shows that contraceptive care and STI care are generally provided separately in Timor-Leste, with contraceptive care almost exclusively positioned within maternity care services, and delivered by midwives. This is reflective of global SRH policy, programming and research, which typically focuses on reproductive health, maternal and child health. While maternal and child health is critical, if implemented with a narrow focus, this approach can come at the expense of sexual health programming [31]. In response, the World Health Organisation (WHO) created operational guidance for SRH, that provides practical guidance about how different components of sexual health and reproductive health programming are conceptually and practically intertwined [31]. Applying this guidance in Timor-Leste is a key next step, including providing more inclusive contraceptive services, and integrating contraceptive and STI care.

The WHO operational guidance also describes the need for a holistic approach to sexual health programming [31]. Although several IDI and PGD participants spoke about the benefits of condom use, most discussion focused on disease transmission, perceived side-effects of condom use, and social harms associated with condom use and sexual relations. For example, there was significant concern amongst participants about condoms negatively impacting sexual pleasure, predominantly for men. This concern has been explored and discussed in other contexts, with evidence showing that people who believe condoms reduce sexual pleasure are less likely to use them [32,33]. While pleasure is a subjective measure, a recent systematic review and meta-analysis indicates that incorporating messaging about sexual pleasure in SRHR programming can improve sexual health outcomes, including condom use [32]. This is strong evidence to include more open and positive discussions about sexual pleasure in SRHR interventions and healthcare provider training. For example, addressing negative and inaccurate beliefs about condom use, promoting pleasure-enhancing elements of condom-use (for example, reduced stress about preventing pregnancy and STI transmission), and discussing ways to make condom use more pleasurable and effective (for example, the use of personal lubricants) [29,32,33]. Inherently, this involves acknowledging that sexual pleasure is one of the key reasons people have sexual relations, and that sexual pleasure and enjoyment is not only for men.

The narrow and overall negative focus on sexual relations and condom use found within our study highlights the stigma and discrimination associated with condom use both within the Timorese health system and broader society. Stigma and discrimination associated with condom use has also been described in other contexts, with similar associations identified between condom use and infidelity, promiscuity and lack of love or trust in a relationship [34–36]. Numerous social harms, such as reputational damage, associated with condom use have been described in other contexts as especially impacting young people and women [35,36]. We found this to be true in our study, with young women and unmarried women facing additional risks of violence if seeking out information and services about condoms, or other SRH needs.

Moreover, there is complexity around the concept of who can use a condom, and who they should use it with. For example, associating condom use with people, especially women, who have been labelled ‘bad’ or ‘dirty’. This indicates significant work is required to challenge and shift harmful social norms, to better enable SRHR and gender equity. Normalising discussions about SRHR and correct condom use will also help respond to misinformation and myths, which is important for improving SRHR outcomes [37].

Changing social norms takes time and strategic investment, at the multiple levels that influence health: individual, family and peers, community and structural (laws, policies, guidelines) [31]. In Timor-Leste, engaging with religious leaders and organisations is vital for increasing access to condoms, as the influence of the Catholic Church on SRH is significant [11,38,39]. The Church’s influence in Timor-Leste is not surprising, given its crucial role in the fight for independence, and its current importance in society, for both spiritual guidance and as a valuable source of information [40]. This influence is further complicated by the Catholic Church’s long and controversial opposition to contraceptive use, including condoms [41]. The varied experiences and opinions about how religious leaders and the church engage with SRHR in Timor-Leste highlights the need for ongoing collaboration between the health sector and this important stakeholder group.

Family planning provider bias is a concern in many different contexts, although the drivers and type of bias varies [7]. Identifying the context-specific causes of family planning provider bias is the first step in being able to adequately respond to it [7]. Our findings indicate that perceived or real provider bias is a significant barrier to the access and uptake of condoms in Timor-Leste, with many socio-cultural and structural drivers identified. Perceived or real provider bias has significant impacts in relation to person-centred contraceptive care and must be addressed if universal access to SRH services is to be achieved [7].

Addressing supply chain challenges is also vital to increase condom access and uptake. While some of the condom distribution issues identified in our study are due to healthcare provider or health facility level challenges (for example, provider bias), the overall insufficient and inconsistent distribution of condom stock suggests supply chain challenges at a national systems level. Strengthening the condom supply chain is important for meeting existing and future demand for condoms and lubricants [29].

At the health facility level, several participants identified creating free and anonymous distribution pathways (for example, having condom dispensers in bathrooms) to increase community access to condoms. There are numerous other examples of successful condom distribution initiatives from around the world that could be usefully adapted for the Timor-Leste context, including interventions that target specific groups, such as young people [42]. Importantly for all contexts, condom supply management and demand generation activities need to be well-coordinated and connected [29].

### Research application

Implemented as operational research, we used our findings to inform and guide our work at MSTL and collaborations with key partners, including the Ministry of Health. This included immediate actions to improve the availability of SRHR information and services for people who want to access condoms now, for example by establishing pathways for people to anonymously access free condoms and lubricants at the MSTL support office and clinic. MSTL has also invested in more long-term responses to help shift harmful social norms and create a more enabling environment for SRHR. For example, insights gained from the research process and findings were used to inform the adaptation of a community-facing social and behaviour change initiative, Stepping Stones, to prevent violence against women and improve SRHR in the Timor-Leste context [43]. Insights from the research were also used to inform health promotion initiatives and program design more broadly, including health sector strengthening initiatives led in partnership with the Ministry of Health. MSTL continues to conduct values-based recruitment of new staff members, and in partnership with the Ministry of Health, invests time and resources into regular values clarification and attitude transformation training of healthcare providers working in SRHR.

### Strengths and limitations

The binary conceptions of sex and gender are limiting and inaccurate, and do not describe the true diversity of people and communities in Timor-Leste. We acknowledge the limitations of using this binary language (male/female and women/men) in our research, along with our focus on sexual relations between heterosexual couples.

We endeavoured to keep the voices of participants central throughout our research work, as demonstrated through our approach to language and translation inclusivity [23]. However, some meaning is still likely to have been lost during translation [44].

Impacts from the COVID-19 pandemic limited planned member checking processes. Only three IDI participants based in Dili were approached and available to engage with and provide feedback on the research transcripts and findings. This was mitigated by inviting other research stakeholders, including healthcare providers who were not participants in the research, to provide feedback during stakeholder engagement activities. We also acknowledge possible power imbalances between researchers and participants, through our associations with a health NGO and an Australian university. We used the ethics of reflexivity and solidarity throughout the research process to help track and explore our own beliefs, positionality, knowledge and assumptions as individuals and as a research team.

## Conclusion

Our research approach and methods used are innovative, rigorous, and centre participant voices. Our findings provide new and contextual understandings about access to condoms in Timor-Leste, that have been used to inform SRH service provision initiatives. Our research approach and findings can be further used to inform wider SRHR review, policy and practice within Timor-Leste, and in other contexts and health areas.

## Supporting information

S1 Appendix Author reflexivity statement. (DOCX)

S2 Appendix Consolidated criteria for reporting qualitative studies (COREQ). (DOCX)

S1 Questionnaire Inclusivity in global research questionnaire. (DOCX)
