# Supplementary material for: ‘It depends on who is asking and why they will use it’: Access to male condoms in Timor-Leste
Source: PLOS Glob Public Health. 2023 Sep 29;3(9):e0002409. doi: 10.1371/journal.pgph.0002409 (PMC10540955; doi:10.1371/journal.pgph.0002409)
Supplement: S1 Appendix — (DOCX) [file pgph.0002409.s001.docx]

**Appendix 1: Reflexivity Statement**

1. **How does this study address local research and policy priorities?**

The overall study was designed as a collaboration between researchers from Marie-Stopes Timor-Leste and The University of Melbourne to address the pressing issue of the high unmet need for family planning and high maternal mortality in Timor-Leste. The overall research design, including research priorities and research questions, was developed collaboratively by the researchers with key Timorese stakeholders, including The National Institute of Health, The Ministry of Health, UNFPA Timor-Leste, The National Midwives Association, The National Network of People Living with HIV in Timor-Leste, The National HIV Commission, The National University of Timor-Leste, HAI International (now known as HAMNASA), JSI, Catalpa, Clinic Café Timor, The Asia Foundation Timor-Leste, and representatives from the Partnership for Human Development (PHD) and The Australian Embassy in Timor-Leste. The field research team (HH, AS, SM, MS, HSX) met with these key stakeholders in Timor-Leste to hear their priorities for health research centred on sexual and reproductive health (SRH), to discuss our ideas and subsequently agree on research priorities and approach. Based on these consultations and following a review of the academic and grey literature in the area of sexual and reproductive health in Timor-Leste, we confirmed our overall research aim, research questions and research objectives. These align with national research priority areas nominated by the Timor-Leste Ministry of Health and previous research, including a focus on and prioritisation of maternal health^[[1]](#footnote-1)^.

1. **How were local researchers involved in study design?**

The study was designed by the research team, in collaboration with key stakeholders. The research team includes five field research team members and three research supervisors. Four of the five field research team members (AS, SM, MS, HSX) are Timorese and come from four different Municipalities in Timor-Leste (Ainaro, Baucau, Bobonaro and Manufahi). Collectively, they speak 16 languages (Bunak, English, Fataluku, Galoli/Galolen, Indonesian, Kairui, Kemak, Lakalei, Lolein, Makalero, Makasai, Mambai, Portuguese, Tetun Prasa, Tetun Terik, Waima'a). Their personal and professional experience and language abilities were pivotal in the design, implementation, analysis and communication of the research findings. One of the three research supervisors (RDA), a highly respected and experienced Timorese public health professional, provided expert guidance and support throughout the research project, including study design. The overall study design was also developed in partnership with local stakeholders (as described in #1).

1. **How has funding been used to support the local research team?**

The five field research team members (HH, AS, SM, MS, HSX) were employed by Marie Stopes Timor-Leste (MSTL) at the time of study design, and during data collection and analysis. MSTL received financial support to conduct this research from the Australian Government Department of Foreign Affairs and Trade. Throughout the study, we have focused on individual and team learning, professional growth and well-being. The research team has conducted peer learning sessions on qualitative research. This included HH facilitating learning sessions with the other field research team members (HSX, SM, AM, MS) about research approaches, reflexivity, data analysis and academic writing skills. AM facilitated team learning sessions about conducting research in Timor-Leste. HSX and SM provided support to other research team members regarding facilitation skills and working with young people. Project funding was also used to facilitate MS and SM co-present research findings at a public health conference in Australia (<https://twitter.com/helrinki/status/1572804464595337216?s=20&t=Jmz4p6fi2vX0mcWHbpAolw> ). While in Australia, MS and SM shared research findings with the MSI Asia-Pacific team, visited MSI Australia clinics and national hotline services, and visited The University of Melbourne. MS has also shared research findings through the MSI Reproductive Choices global partnership. Moreover, time and project funding was allocated throughout the research process to support de-briefing, reflection and wellness activities amongst the Timorese field research team.

1. **How are research staff who conducted data collection acknowledged?**

The five field research team members responsible for data collection (HH, HSX, SM, AM, MS) are all co-authors of this research paper. Other MSTL colleagues who supported data collection logistics (for example, driving the researchers to data collection sites, and preparing data collection finances) have been acknowledged in our research paper.

1. **Do all members of the research partnership have access to study data?**

Yes.

1. **How was data used to develop analytical skills within the partnership?**

The research team worked collaboratively to leverage their multidisciplinary skill sets

during data analysis and across the whole study. More formally, HH conducted learning sessions about data collection, data analysis, data interpretation, data presentation, and scientific writing as part of the research partnership to strengthen the analytic and writing skills of the other field research team members (see #3).

1. **How have research partners collaborated in interpreting study data?**

As mentioned in #3 and #6, the research team has worked collaboratively to interpret study data. Preliminary research findings were presented to The National Institute of Health for input and insight. Preliminary research findings were also shared and discussed with key stakeholders before being finalised, including with health care providers in Timor-Leste, and representatives from The Ministry of Health, The National University of Timor-Leste, Catalpa International, The Asia Foundation, PHD and The Australian Embassy in Timor-Leste. Preliminary findings were also shared widely within Marie Stopes Timor-Leste to gain feedback on the analysis process and findings gained. The field research team of five has worked together to understand and co-develop the implications for research, policy and practice, under the supervision of the three senior experienced research supervisors.

1. **How were research partners supported to develop writing skills?**

The research team writing this paper is a mix of senior and junior academics, public health practitioners and clinicians. MAB and CV have completed their PhDs in the last 15 years and have significant experience conducting and teaching health research and writing at a Tertiary level. RDA is a senior public health leader in Timor-Leste, with extensive experience in health policy, practice, and research. The authorship team was supported by MAB, CV, and RDA throughout the research process. HH is currently working on her PhD, and supported other field research team members to develop and refine writing skills, including through team learning sessions about scientific writing (see #3), extensive review and feedback sessions on draft documents, and regular zoom and WhatsApp calls. Key research documents (for example, the development of a plain language research report) were shared with core research partners for feedback and buy-in on both content and writing style.

1. **How will research products be shared to address local needs?**

All papers resulting from our research to date have been published as open access. The abstract or full paper of each publication has been translated into one of the National languages in Timor-Leste, Tetun, and made publicly available online. Research findings were presented to the ethics committee at the National Health Institute of Timor-Leste (INS). We have produced a plain language research report in English and Tetun that are publicly available online. Printed copies of the plain language research report were distributed to key partners (including to INS and The Ministry of Health) and are available to the general public through the MSTL office and outreach services.

MSTL staff have verbally shared findings within Timor-Leste, including with the Ministry of Health, study participants and communities involved in the study.

1. **How is the leadership, contribution and ownership of this work by LMIC researchers recognised within the authorship?**

Five of the eight co-authors (RDA, AS, MS, SM, HSX) are researchers from Timor-Leste. RDA is one of three research supervisors overseeing the overall implementation of the project.

1. **How have early career researchers across the partnership been included within the authorship team?**

The authorship team is primarily composed of early career researchers (HH, AS, SM, HSX, MS). They have been involved in conducting all stages of the study. We acknowledge that one of the early career researchers (HH) is from a high-income country (Australia) and the other four early career researchers (AS, SM, HSX, MS) are from Timor-Leste.

1. **How has gender balance been addressed within the authorship?**

Five authors are female (HH, AS, SM, CV, MAB) and three are male (MS, HSX, RDA).

1. **How has the project contributed to training of LMIC researchers?**

Please refer to #3 about the specifically designed peer learning and training workshops with early career LMIC researchers (AS, SM, MS, HSX) in qualitative research data collection, validation, analysis, and writing. Two members of the early career research team (MS, SM) travelled to Australia to co-present our research findings at the Population Health Congress in 2022. During this trip, MS and SM were able to participate in training made available through the conference, and spend time at The University of Melbourne, MSI Asia-Pacific and MSI Australia clinical sites.

1. **How has the project contributed to improvements in local infrastructure?**

MSTL is a major provider of Sexual and Reproductive Health Services in Timor-Leste, working in partnership with the Timor-Leste Ministry of Health. This project was designed as operational research and has directly fed into the design and delivery of health services by Marie Stopes Timor-Leste.

1. **What safeguarding procedures were used to protect local study participants and researchers?**

We adhered to Marie Stopes Timor-Leste safeguarding policies and practices throughout the research process, which are aligned with National and International best practice standards. Regular debriefing and reflexive discussions occurred within the research team to help identify and address any issues arising during the study – this process of reflection during data collection is critical to any research involving sensitive topics or violence to ensure the safeguarding of research participants and research teams. The National Health Institute of Timor-Leste ethics board conducted routine quality monitoring of the field research on two separate trips, observing three PGDs and two IDIs in two different municipalities (Bobonaro and Manufahi).

1. Ministry of Health, National Strategy on Reproductive, Maternal, Newborn, Child and Adolescent Health, 2015-2019. Timor-Leste <https://srhr.org/abortionpolicies/documents/countries/04-Timor-Leste-National-Strategy-on-ReproductiveMaternal-Newborn-Child-and-Adolescent-Health-Ministry-of-Health-2015.pdf> [↑](#footnote-ref-1)
