# Supplementary material for: ‘It depends on who is asking and why they will use it’: Access to male condoms in Timor-Leste
Source: PLOS Glob Public Health. 2023 Sep 29;3(9):e0002409. doi: 10.1371/journal.pgph.0002409 (PMC10540955; doi:10.1371/journal.pgph.0002409)
Supplement: S1 Questionnaire — (DOCX) [file pgph.0002409.s003.docx]

Inclusivity in global research

PLOS’ policy on inclusivity in global research aims to improve transparency in the reporting of research performed outside of researchers’ own country or community and ensures that PLOS publications reporting global research adhere to high standards for research ethics and authorship. Authors of relevant research articles may be asked to complete the questionnaire below, which outlines ethical, cultural, and scientific considerations specific to inclusivity in global research. This questionnaire may be requested when researchers have travelled to a different country to conduct research, if research uses samples collected in another country, research with Indigenous populations or their lands, or if research is on cultural artefacts. Researchers travelling to another country solely to use laboratory equipment will not normally be required to complete the questionnaire. However, the questionnaire can be requested at the journal’s discretion for any submission – if you have been requested to complete this questionnaire by the PLOS journal you submitted to, please do so.

Please complete the questionnaire below and include this as a Supporting Information file with your manuscript. Note that if your paper is accepted for publication, this checklist will be published with your article in the supporting information files. Please ensure that you reference the checklist in the main body of your manuscript. We suggest adding a subsection ‘Inclusivity in global research’ to your Methods section and adding the following sentence: “Additional information regarding the ethical, cultural, and scientific considerations specific to inclusivity in global research is included in the Supporting Information (SX Checklist)”

The questions have been designed to be applicable to a wide range of study types, and there are subsections for both human subjects research and non-human subjects research. If any of the questions are not relevant to your research please mark them as “N/A” as appropriate.

**Ethical considerations, permits and authorship**

*This section is applicable to all research types.*

Provide details as to who granted permissions and/or consent for the study to take place in the Methods section of your manuscript. This should include the names of **all** ethics boards, governmental organizations, community leaders or other bodies that provided approval for the study. If individuals provided approval refer to these people by their role or title but do not list their name(s).

Reported on page number: 8,9,10

If there were any deviations from the study protocol after approval was obtained please provide details of these changes in the Methods section of your manuscript.
Did this study involve local collaborators that are residents of the country where the research was conducted or members of the community studied? If you do not have any authors from said communities, please provide an explanation for this below.

Reported on page number: N/A

Five of the eight authors are from Timor-Leste. This includes four of the five field research team members (AS, SM, MS, HSX), who come from four different Municipalities in Timor-Leste (Ainaro, Baucau, Bobonaro and Manufahi). One of the three research supervisors (RDA) is a highly respected and experienced Timorese medical professional and heath leader.

Everyone listed as an author should meet PLOS’ criteria for authorship and all individuals who meet these criteria should be included in the author byline, rather than the acknowledgements. Authorship criteria is based on the International Committee of Medical Journal Editors (ICMJE) Uniform Requirements for Manuscripts Submitted to Biomedical Journals - for further information please see here: <https://journals.plos.org/plosone/s/authorship>.

**Human subjects research (e.g. health research, medical research, cross-cultural psychology)**

Did you obtain written informed consent from a representative of the local community or region before the research took place? How did you establish who speaks for the community? Details of written informed consent obtained from study participants should be reported separately in the Methods section of your manuscript.

Ethics approval was provided by the National Health Institute of Timor-Leste (1168MS-INS/DE/DEP/V112019). For participatory group discussions, we coordinated with local community leaders before the commencement of data collection. This included sending letters, in the national language Tetun, with information about the research process. We verbally spoke to these community leaders over the phone or in person, to discuss what was written in the letter and to answer any questions they had. For in-depth interviews with healthcare providers, we coordinated with Municipality Health Directors from each study municipality before commencing data collection, to facilitate communication with facility-level health directors and healthcare providers.

How did members of the local community provide input on the aims of the research investigation, its methodology, and its anticipated outcome(s)?

Our study was implemented in collaboration between Marie Stopes Timor-Leste (MSTL), an SRH specialist organisation working in partnership with the Timor-Leste Ministry of Health, and The University of Melbourne. At the time of research design and data collection, the five field research team members (HH, MS, HSX, SM, AS) were employed by MSTL in health program, management and service delivery roles. We developed research questions and methodology in collaboration with key stakeholders, including the National Health Institute of Timor-Leste, healthcare providers, and healthcare users in Timor-Leste. An author reflexivity statement reflecting on the dynamics of our research team and collaboration process is available in Appendix 1.

When engaging with the local community, how did you ensure that the informed consent documents and other materials could be understood by local stakeholders?

Participant consent forms and a plain language research statement were designed and piloted in the national language Tetun, for accuracy and understanding. The final versions of these documents were approved by three different ethics committees. These documents were provided to all research participants. We carefully explained the difference between research and education activities, to help people with no prior research experience understand what we would be doing before agreeing to participate or not. Written consent (in Tetun) and verbal informed consent (in the language preference of the participant) was provided by all participants. A total of 13 different languages were used by participants: Tetun, Portuguese, Indonesian, English, Tetun Terik, Baikenu, Bunak, Fataluku, Kairui, Kemak, Lakalei, Makasai, and Mambai. We write about our use of language inclusivity in the following publication:

Henderson, H., Marques da Silva, A., et al (2022). Participatory qualitative research in a multilingual context: the use of panel translation to better understand and improve sexual and reproductive health in Timor-Leste. *Qualitative Health Research, 32*(10), 1498 - 1513. doi:<https://doi.org/10.1177/10497323221110800>

Will the findings of the research be made available in an understandable format to stakeholders in the community where the study was conducted (e.g. via a presentation, summary report, copies of publications, etc.)? Please provide details of how this will be achieved.

A plain language research report was produced in English and Tetun. Hard copies were shared with key stakeholders, including the National Health Insitutute of Timor-Leste and the Ministry of Health. These reports are publicly available online: <https://www.mariestopes.tl/resources/>. Research findings were shared verbally in the national languages of participants, through municipality-based educators and team members, employed by Marie Stopes Timor-Leste. A formal presentation of research findings was presented to the National Health Insitute of Timor-Leste. One peer-reviewed publication about research methods has been translated in full into Tetun. This is available online: <https://www.researchgate.net/profile/Helen-Henderson-3>. At a minimum, the abstracts of all future peer-reviewed publications will be translated into the Tetun. Research findings will be presented verbally in Tetun at the 2023 Timor-Leste Studies Association Research Conference: <https://tlstudies.org/conference/>

**Non-human subjects research using specimens/ animals collected as part of the study, or those housed in archival collections. Examples include archaeology, paleontology, botany and zoology.**

Did the permission you obtained from a local authority to perform the study include an agreement on access to outputs and benefit sharing? This may include procedures to enable fair distribution of the benefits and resources arising from the research performed. Please include any details of Prior Informed Consent and Benefit Sharing Agreements obtained. These may be required by field-specific regulations, for example the Convention on Biological Diversity (CBD) and the associated Nagoya Protocol.

Ethics approval from the National Health Institute of Timor-Leste required us to share research findings verbally and in a report before being publicly released. This was completed.

If the material used in your study was imported, please A) provide the year it was imported and B) indicate whether permits were obtained to import/export the materials used, C) provide details of any permits obtained. If this information is not available, please indicate this.

N/A – no archival material was imported/exported.

If you used archival specimens, please state how the material used in your study was acquired by the institute it is held in and provide details of any permits obtained for the original excavations/ sample collection. If this information is not available, please indicate this.

N/A – no archival speciments were used in this study.

How was the potential cultural significance of the materials collected in your study to local communities considered in your research design? Were Indigenous peoples and/or local researchers and institutions involved with archaeological excavations / collection of specimens? If so, please provide a description of their involvement.

N/A – no materials or specifimens were collected in this study.

If your manuscript includes photographs of human remains please indicate whether authors obtained permission from descendants or affiliated cultural communities to do so.

N/A – no human remains were part of this study.
